# Biomechanics and Load Resistance of Short Dental Implants: A Review of the Literature

**DOI:** 10.1155/2013/424592

**Published:** 2013-05-08

**Authors:** Istabrak Hasan, Christoph Bourauel, Torsten Mundt, Friedhelm Heinemann

**Affiliations:** ^1^Endowed Chair of Oral Technology, Department of Prosthodontics, Preclinical Education and Dental Materials Science, Rheinische Friedrich-Wilhelms University, Welschnonnenstraße 17, 53111 Bonn, Germany; ^2^Endowed Chair of Oral Technology, Rheinische Friedrich-Wilhelms University, Welschnonnenstraße 17, 53111 Bonn, Germany; ^3^Department of Prosthodontics, Gerodontology and Biomaterials, University of Greifswald, Walther-Rathenau-Strasse 42a, 17475 Greifswald, Germany

## Abstract

This paper was aimed to review the studies published about short dental implants. In the focus were the works that investigated the effect of biting forces of the rate of marginal bone resorption around short implants and their survival rates. Bone deformation defined by strain was obviously higher around short implants than the conventional ones. The clinical outcomes of 6 mm short implants after 2 years showed a survival rate of 94% to 95% and lower survival rate (<80%) for 7 mm short implants after 3 to 6 years for single crown restorations. The short implants used for supporting fixed partial prostheses had a survival rate of 98.9%. Short implants can be considered as a good alternative implant therapy to support single crown or partial fixed restorations.

## 1. Introduction

After tooth loss, severely atrophic residual alveolar ridges are fairly common, especially in patients who have been edentulous for a long period of time. The bone volume of posterior areas of the maxilla and the mandible is frequently insufficient for the placement of implants with adequate dimensions, unless a procedure such as ridge augmentation or sinus floor elevation is performed. Although widely utilised, these techniques imply greater morbidity, longer treatment times, and higher costs. The sinus cavity in the maxilla and alveolar nerve proximity in the mandible are clinical situations where short implants could be considered as an alternative treatment option. 

Some have hesitated to use these implants due to the perception of a higher risk of failure compared with longer implants for both fixed restorations [1–5], as well as maxillary overdentures [6, 7]. More recent studies, however, suggested that short implants (7 to <10 mm) can reach similar success rates as longer ones for the support of fixed partial dental prostheses [8–10]. Even 3-year [11] and 7-year [12] followup studies reported retrospectively that short implants (8 to 9 mm long) [9, 13, 14] were not less successful compared with implants >10 mm long in the posterior region with fixed partial dental prostheses.

This paper was aimed to review the works regarding the stability and survival rate of short implants under functional loads. Numerical and clinical studies were reviewed.

## 2. Implant Fatigue under Biting Forces 

Prospective studies have shown the positive effect of conventional implant therapy on maximum bite force [15–19]. However, alveolar bone (similar to long bones) adapts its strength to the applied mechanical loading by means of bone modelling/remodelling [20–22]. The response to increased mechanical stress beyond a certain threshold produces fatigue microdamage resulting in bone resorption [23]. The type of attachment system provides different degrees of horizontal and vertical resistances against dislodging forces that could lead to different magnitudes of loading transmission to the implant-bone interface. This does not seem to evoke bone resorption around conventional implants [24, 25]. 

The initial evidence that suggests high predictability of short implants has been reinforced by the different biomechanical studies. It was addressed that maximum bone stress is practically independent of implant length [26] and even that implant width is more important than the additional length [27]. Based on these data, it is believed that with an optimised implant design and surgical protocol, short implants may play an outstanding role in oral implantology, reducing the indications for such procedures as sinus lift and additional grafting techniques [28].

Hasan et al. [29] and Bourauel et al. [30] analysed numerically eight commercial short implants in posterior bone segments and investigated in the osseointegrated state under static occlusal force of 300 N. Implant diameter and geometry had a pronounced effect on stresses in the cortical plate. Strain values obtained with the short implants were drastically higher (clearly above 10,000 *μ*strain) in comparison to long implants (5,000 *μ*strain, in general).

Rossi et al. [31] evaluated prospectively the clinical and radiographic outcomes of 40 implants (SLActive, Straumann) with a length of 6 mm and moderately rough surface supporting single crowns in the posterior regions. The implants were loaded after 6 weeks of healing. Implant survival rate, marginal bone loss, and resonance frequency analysis (RFA) were evaluated at different intervals. The clinical crown/implant ratio was also calculated. They obtained a survival rate of 95% before loading. No further technical or biological complications were encountered during the 2-year followup. The mean marginal bone loss before loading was 0.34 to 0.38 mm. After loading, the mean marginal bone loss was 0.23 to 0.33 and 0.21 to 0.39 mm at the 1-year and 2-year followups. They reported that clinical crown/implant ratio increased with time from 1.5 at the delivery of the prosthesis to 1.8 after 2 years of loading.

Arlin [32] reported that a success rate of 94% for 6 mm Straumann implants with a moderately rough surface was after 2 years of loading. However, lower success rate (<80%) was presented for 7 mm implants with a machined surface after 3 and up to 6 years of followup [31, 33, 34].

## 3. Short Implants for Supporting Prostheses

Van Assche et al. [14] investigated the outcome of short implants additionally placed with longer implants to support a maxillary overdenture. Twelve patients received six implants to support a maxillary overdenture. They concluded that an overdenture on six implants, of which two have a reduced length, might represent a successful treatment option. The study showed no significant difference in both implant lengths at 2-years followup. 

The retrospective study of Anitua and Orive [28] showed that the overall survival rates of 1,287 short implants (<8.5 mm) in a mean followup period of 47.9 to 24.46 months were 99.3% and 98.8% for the implant and subject-based analysis, respectively. They suggested that treatment with short implants can be considered safe and predictable if they are used under strict clinical protocols.

Misch et al. [35] evaluated implant survival when a biomechanical approach was used to decrease stress to the bone-implant interface. They underwent a retrospective evaluation of 273 consecutive posterior partially edentulous patients treated with 745 implants, 7 or 9 mm long, supporting 338 restorations over a 1-year to 5-year period. Implant survival data were collected relative to stage I to stage II healing, stage II to prosthesis delivery, and prosthesis delivery to as long as 6-year followup. A biomechanical approach to decrease stress to the posterior implants included splinting together with no cantilever load, restoring the patient with a mutually protected or canine guidance occlusion, and selecting an implant designed to increase bone-implant contact surface area. A 98.9% survival rate was obtained from stage I surgery to prosthetic followup. 

Griffin and Cheung [8] studied retrospectively the success rate of 168 hydroxyapatite- (HA-) coated short implants (6 mm diameter, 8 mm length) placed in mandibular and maxillary molar areas with reduced bone height. There were 128 implant-supported single crowns. Thirty-eight implants served as abutments for fixed partial dentures connected to other implants of various sizes. Two implants were involved in cantilevered fixed partial dentures. Patients were followed for up to 68 months after loading of implants. The overall cumulative success rate was found to be 100%.

Yang et al. [36] evaluated experimentally the biomechanical performance of seven 7 mm short implants in splinted restorations using strain gauges. The implants were splinted together (short-short implant splinted restoration, SS) or individually with a 4.4 × 12.0 mm implant (short-long implant splinted restoration, SL), and a 50 N oblique load was applied to both restorations. They observed that the strain was significantly decreasing with increasing implant diameter in both the SS and SL restorations, and the observed strain was identical for the splinted implants of the same diameter and those splinted to the long implant. They suggested that splinting of two short implants has the same biomechanical effectiveness as splinting to a single long implant.

Pieri et al. [37] evaluated prospectively the clinical and radiographic outcomes of 61 submerged ultrashort implants (4 mm diameter, 6 mm length) supporting fixed partial dentures in severely atrophic posterior mandibles. The implants were loaded after 5 to 6 months. They recorded a failure of two implants before loading, while the other implants had favourable clinical and radiographic findings throughout the observation period (2-year survival and success rate: 96.8%). Mean changes in marginal bone levels were stable (0.40 ± 0.23, 0.51 ± 0.38, and 0.60 ± 0.13 mm after 6 months and 1 and 2 years, resp.) and were unaffected by measured crown-to-implant ratios (range: 1.31 to 3.12). An overview of the survival rate is presented in Table 1.

## 4. Clinical Indications of Short Dental Implants

Main indication for the short implants is in the posterior upper and lower jaw where there is extreme residual bone resorption above the maxillary sinus and the mandibular. 

In cases of fixed implant-supported restorations of edentulous jaws, one alternative to short implants is to omit implants in posterior jaw and provide a cantilever solution. Clinical studies reported this treatment option as a reliable and successful solution [38, 39]. However, when there is sufficient residual bone and for cost reasons, additional short implants are to be inserted to provide an additional support in the distal region [35].

Principally, in all cases of reduced residual bone height in the posterior region, augmentation procedures are alternatively used to ensure the placement of conventional length implants. In the upper jaw, this can be provided by sinus augmentation techniques. These proceedings imply either crestal approach or a lateral window approach, normally including a more extensive procedure corresponding postoperative pain and swelling [40]. 

For the crestal approach, the sinus membrane is pushed higher with punch and mallet or nowadays also with piezo-surgery devices. Lateral window approach includes a gingival flap design of the lateral alveolar crest and careful ablation of the bone until mucous membrane is reached. Thereafter, the soft tissue is lifted slowly and cautiously with special sinus lifting instruments until the lateral nasal wall is reached. Specific care need to be paid whenever a sinus septum is contained in the sinus. Main complication of the procedure is the infraction and if not detected consequent leakage of the membrane and possible sinus infection, which require a complicated long-winded treatment [41, 42]. There was no statistically significant difference between one or the other sinus lifting technique or the placement of shorter implants too [43–45].

When the height of the alveolar bone in lateral side of the mandible is not sufficient for conventional implant length, bone augmentation, as alternative to short implants, is definitely more complicated and less predictable than bone augmentation in the sinus area [46]. Moreover, short implants in this region are an interesting alternative and a therapeutical option to vertical augmentation since the treatment is faster, cheaper, and associated with less morbidity [47]. When height limitation is not considered properly and a longer implant is chosen, the supplying nerve may be injured. Injury of the mental nerve is one of the main complications in dental implantology. The incidence of transient altered lip sensations was noted by several investigators from 8.5% to 24% of patients [48]. Greenstein and Tarnow [48] reported that the guidelines for implant placement include leaving a 2 mm safety zone between an implant and the coronal aspect of the nerve. Therefore, the observation of the inferior alveolar nerve and mental foramen on panoramic and periapical films prior to implant placement is essential. Other authors agree with Greenstein to maintain a spatial distance of 2 mm or more for safety reasons in three dimensional planning [49, 50]. 

## 5. Conclusions

The selection of dental implants is a critical issue that strongly affects the final functional and aesthetic results. The choice of implant length in relation to the available bone quality and biting force is an essential factor in deciding the survival rates of these implants and the overall success of the prosthesis. Short implants offer the possibility to avoid bone augmentation for the patients with advanced alveolar bone resorption, where the insertion of regular-length dental implants (>8 mm) is problematic. In particular, in the posterior mandibular and maxillary regions, where there is a risk of injuring the inferior alveolar nerve or penetrating the maxillary sinus during implant placement when alveolar bone is deficient.

By considering the biomechanical aspect of short implants, the reviewed studies showed a high survival rate for short implants and comparable marginal bone resorption to the conventional implants for a period from 2 to 3 years. Short implants can be a successful alternative to bone augmentation techniques. However, special consideration have to be taken to optimise the occlusion of the final restoration and to avoid the lateral loading of the implants that caused by the improper occlusal relation. There is, however, the lack of long-term clinical studies. Such studies are essential since the experimental and numerical investigations showed a relative high strain of the bone bed around short implants in comparison to the conventional implants.

## Figures and Tables

**Table 1:** Overview of the reviewed studies and the obtained survival rates.

Study	Number and length of implants	Restoration type	Study length	Survival rate
Rossi et al. [31]	40, 6 mm	Single crowns	2 years	95%
Arlin [32]	630, 6 mm	—	2 years	94%
Van Assche et al. [14]	36, 6 mm	Maxillary overdenture	2 years	99%
Anitua and Orive [28]	1,287, <8.5 mm	—	47.9–24.46 months	99.3%–98.8%
Misch et al. [35]	745, 7 and 9 mm	—	6 years	98.9%
Griffin and Cheung [8]	168, 6 and 8 mm	Single crowns and fixed partial dentures	68 months	100%
Pieri et al. [37]	61, 4 and 6 mm	Fixed partial dentures in mandible	2 years	96.8%
